# The “pro-inflammatory imprint” of ultra-processed food exposure: a review of mechanisms affecting child and adolescent health and development via the gut microbiome-inflammation axis

**DOI:** 10.3389/fnut.2026.1843587

**Published:** 2026-09-16

**Authors:** Qinghua Liu, Lijiao Luo, Zhiwen Yan, Pan Yang, Teng Long, Yuntao Yang, Min He, Li Tao

**Affiliations:** 1 College of Bioinformatics Science and Technology, Harbin Medical University, Harbin, China; 2 College of Life Science and Technology, Huazhong Agricultural University, Wuhan, China; 3 Department of Neurology, The First Affiliated Hospital of Chongqing Medical University, Chongqing, China; 4 Department of Clinical Nutrition, People's Hospital of Chongqing, Hechuan, Chongqing, China

**Keywords:** childhood obesity, Dietary Inflammatory Index (DII), food environment, gut-brain axis, metabolic programming, neurodevelopment, ultra-processed foods

## Abstract

The prevalence of ultra-processed food (UPF) consumption in pediatric diets has raised significant concerns regarding long-term health. This review examines evidence for a proposed ‘pro-inflammatory imprint' involving the gut microbiome–inflammation axis. Using a structured narrative synthesis of pediatric observational evidence, adult intervention studies, preclinical research, multi-omics data, and public health evaluations, we examine associations between UPF exposure and metabolic, inflammatory, cognitive, and neurodevelopmental outcomes while distinguishing direct from indirect evidence. We propose the Pediatric-Specific Integrated Dietary Inflammatory Index (cP-IDII) as an unvalidated, hypothesis-generating research framework for integrating reproducible nutrient- and processing-related dietary exposures; downstream microbial, intestinal-barrier, inflammatory, metabolic, and immune measures are reserved for external validation. Furthermore, we discuss the role of commercial determinants and environmental factors in shaping child health. The synthesis identifies measurement gaps and complementary policy entry points across product composition, food environments, affordability, and marketing. Empirical development and external validation are required before the cP-IDII can be used for clinical, epidemiological, or public health decision-making.

## 1 Introduction

Over the past half-century, the prevalence of overweight and obesity among children and adolescents (aged 5–19 years) has exhibited a substantial rise, currently affecting hundreds of millions of individuals globally (1). Pediatric overweight and obesity are multifactorial outcomes shaped by interactions among energy intake, dietary quality, physical activity, sleep, socioeconomic conditions, and other environmental influences. UPFs may facilitate excess energy intake because many products are energy-dense, highly palatable, rapidly consumed, high in free sugars, sodium, or unhealthy fats, and low in dietary fiber (2). This is further compounded by the complex interplay between overall environmental factors (e.g., air pollution, endocrine disruptors, and sedentary behaviors) and gene expression (3, 4). Early life stages, encompassing intrauterine development, infancy, and adolescence, serve as pivotal periods for metabolic programming, gut microbiome colonization, and central nervous system maturation. During these periods, UPF exposure may alter gene expression and immune function, shaping long-term health trajectories, particularly concerning metabolic and neurodevelopmental processes (5, 6).

Potential age-specific susceptibility should not be treated as a uniform property of all children. Infancy includes rapid gut microbiome assembly and maturation of intestinal and immune functions; early childhood includes continued adipose-tissue development and metabolic programming; and childhood and adolescence involve ongoing synaptic remodeling, myelination, and epigenomic regulation. These processes provide biological plausibility for age-dependent effects, but direct pediatric evidence linking specific UPF components to these pathways remains limited. Emerging epigenome-wide association studies (EWAS) report associations between childhood UPF consumption and DNA methylation differences (e.g., at loci associated with thyroid hormone and liver function), suggesting that UPF exposure could leave a lasting “imprint” at the transcriptomic level (7). Chronic, low-grade systemic inflammation has been proposed as a potential mechanistic link between high UPF intake and adverse developmental outcomes. This hypothesis is supported by observed associations with early cardiometabolic risk markers (such as adiposity and elevated blood pressure) (8–10) and may be relevant to subsequent health trajectories across the lifespan. The Dietary Inflammatory Index (DII) and its pediatric-specific adaptation (C-DII) (11), alongside the Empirical Dietary Inflammatory Pattern (EDIP), are widely utilized to quantify the overall pro-inflammatory potential of diets (12). Recent longitudinal analyses of US adolescents report that moderate-to-high pro-inflammatory dietary scores are associated with a higher likelihood of prediabetes (13). Although recent meta-analyses report associations between higher DII scores and depression, anxiety, and other neuropsychiatric outcomes (14, 15), research on pediatric populations—especially integrating gut microbiome succession and neurodevelopmental trajectories—remains fragmented, with most existing UPF literature focusing on adult cardiovascular disease.

The DII estimates the inflammatory potential of dietary components, the C-DII adapts this approach for pediatric dietary assessment, and the EDIP is an empirically derived dietary-pattern score. NOVA characterizes the extent and purpose of food processing, whereas the DI-GM emphasizes dietary features associated with microbiome support. These approaches cover complementary dimensions but do not provide a validated pediatric measure that integrates reproducible nutrient- and processing-related exposures while keeping downstream biological responses separate from the exposure score. The cP-IDII is therefore introduced as an unvalidated, hypothesis-generating framework and research agenda rather than as a tool with demonstrated predictive capacity.

This structured narrative review uses life-course and exposome perspectives to organize evidence by exposure, outcome, candidate pathway, and measurement gap. A meet-in-the-middle perspective is used to distinguish external dietary exposures from candidate intermediate molecular changes, including altered branched-chain amino acid metabolism (16). Furthermore, this review evaluates whether these pathways are biologically plausible without presenting microbiome changes, barrier dysfunction, inflammation, or BCAA metabolism as an established continuous causal chain in children. In parallel, by contextualizing the global public health landscape, this review discusses how commercial marketing and underlying demographic determinants (such as education level and socioeconomic status) (17, 18) may shape exposure within a socio-ecological system, thereby identifying potential and complementary policy entry points.

## 2 Review approach

We conducted a structured narrative search of PubMed and Embase from database inception to May 2026. Medical Subject Headings and free-text terms related to ultra-processed foods, NOVA classification, dietary inflammation, pediatric populations, gut microbiome and intestinal barrier function, metabolic and neurodevelopmental outcomes, commercial determinants of health, food environments, and policy interventions were combined using Boolean operators. A representative search strategy was: (“ultra-processed food” OR “ultraprocessed food” OR UPF OR NOVA OR “dietary inflammatory index”) AND (infant OR child OR adolescent OR pediatric OR pregnancy) AND (inflammation OR microbiome OR “intestinal permeability” OR obesity OR metabolic OR neurodevelopment OR cognition OR mental health OR marketing OR “food environment” OR policy). Reference lists of key reviews and eligible studies were also screened, and targeted searches were conducted for specific additives, biomarkers, dietary indices, and policy interventions.

Peer-reviewed human studies involving participants aged 0–19 years were prioritized when they assessed UPF exposure, dietary inflammatory potential, relevant health outcomes, or candidate intermediate pathways. Prenatal exposures were considered separately from children's own dietary intake. Studies without relevant dietary exposures or outcomes and non-peer-reviewed reports were excluded from the evidence synthesis, although market audits were retained when used only to describe product composition or marketing. Adult intervention studies and preclinical studies were included solely to assess mechanistic plausibility.

Evidence was prioritized by directness to the pediatric population and by study design. Priority was given to pediatric systematic reviews and meta-analyses, longitudinal cohorts, randomized or quasi-experimental studies, and well-characterized pediatric observational cohorts. Adult and preclinical evidence was considered indirect and was used primarily when direct pediatric mechanistic evidence was unavailable. Conflicting findings were considered in relation to population age, exposure and outcome definitions, dietary assessment, sequencing or biomarker methods, and risk of confounding. This approach supports a structured narrative synthesis; it is not a systematic review or meta-analysis, and no PRISMA-style study-count inference is intended.

## 3 Ultra-processed foods: definition, characteristics of pediatric exposure, and environmental drivers

### 3.1 Pediatric-specific challenges based on the NOVA classification

The NOVA classification is an internationally recognized framework that categorizes foods according to the extent and purpose of industrial processing. It defines UPFs as formulations of ingredients created through a series of industrial techniques, typically containing cosmetic food additives (e.g., colorants, emulsifiers, sweeteners) and industrial ingredients rarely used in domestic kitchens (e.g., modified starches, hydrogenated oils) (19). Although NOVA is widely used in epidemiology, it describes processing-related characteristics and does not itself demonstrate pediatric-specific biological vulnerability or causal effects. Market audits of commercial complementary foods in France, Portugal, and Australia indicate that between 61.8 and 87.9% of packaged products targeting infants and toddlers under 36 months are classified as UPFs. Many contain free sugars or concentrated fruit juices while also carrying claims such as “no added sugar” (20–22). These market data describe the composition and promotion of products available during a period when food preferences develop, but they do not establish developmental harm. Prospective studies are needed to determine whether early exposure influences later preferences or metabolic outcomes.

### 3.2 Nutritional transition and the globalization of the double burden of malnutrition

UPF consumption is accelerating globally. In the US and the UK, UPFs account for over 67 and 60% of total daily energy intake among children and adolescents, respectively (21, 23). Eliminating UPFs from UK pediatric diets could potentially reduce the prevalence of excess free sugar consumption by 47% (21). UPF sales and intake are also increasing in many low- and middle-income countries undergoing nutrition transition. In Africa and Latin America, urbanization and large-scale investments by transnational food corporations have coincided with increased UPF and sugar-sweetened beverage (SSB) sales (24, 25). An ecological time-series analysis across nine African nations reported an ecological association between increasing SSB sales and adult type 2 diabetes burden (25). The multicountry European I. Family study similarly reported that UPFs contributed 50%−70% of daily total fat, saturated fat, and sugar intake among youth and that higher UPF intake was associated with lower overall diet quality (measured by HDAS scores) (26).

This nutritional shift is equally critical in Asia. In China, over 60% of pre-packaged foods are UPFs, routinely exceeding free sugar and sodium limits across various nutrient profiling models (NPMs) (27). In India, pediatric associations have introduced the “JUNCS” framework (comprising junk foods, UPFs, nutritionally inappropriate foods, caffeinated drinks, and SSBs) to address the childhood obesity epidemic (28). In Mexico and Brazil, escalating UPF intake has been examined alongside obesity trends and the “Double Burden of Malnutrition” (DBM)—the coexistence of undernutrition and overnutrition within the same population or household (29, 30). Particularly among families with low socioeconomic status (SES), structural barriers and health inequities position UPFs as an accessible, cheap caloric source, and may contribute to persistent nutritional inequities (29, 31).

### 3.3 Commercial, household, and socio-ecological determinants of pediatric UPF exposure

Pediatric UPF consumption is shaped by a socio-ecological system in which caregivers' purchasing and feeding decisions reflect household resources, time, food prices and availability, cultural practices, marketing, institutional settings, and public policy (32). Commercial determinants, including product formulation, pricing, placement, and child-directed marketing, constitute one set of influences within this multilevel system rather than its sole cause (32).

Packaging and Cartoon Marketing: Market surveys in Latin America and Europe reveal that a substantial proportion of UPF packaging utilizes cartoons, brand mascots, and toy bundling to captivate pediatric demographics (21, 33, 34). An 11-country review in Latin America found that over 60% of food marketing aimed at youth promoted unhealthy UPFs, heavily relying on licensed characters (35). Furthermore, the industry frequently utilizes health-oriented claims and imagery—such as “free from” claims or realistic imagery of whole fruits and grains—which can influence parents' perceptions and purchase intentions for these snacks (36, 37). This strategy not only increases purchasing rates but also may shape familiarity with and preferences to high-sugar and high-salt foods among children (38).

Digital and Social Media Advertising: The social media industry itself functions as a CDoH shaping the digital food environment (39). Fast-food and UPF brands use celebrity endorsements, price promotions, and emotional appeals across platforms (e.g., YouTube, Facebook, Instagram) (40, 41). Studies in Ghana and Argentina note that children are frequently exposed to UPF advertisements during prime-time television, with an almost total absence of healthy food promotion (42–45). Recently, covert marketing via “Kid YouTubers” has proliferated; UPF product placements in these videos are highly frequent and generate deeper engagement than traditional channels, creating a poorly regulated hybrid media format (46, 47). Experimental and observational evidence suggests that repeated food-marketing exposure may influence reward anticipation and food choice, although causal and long-term pediatric effects remain uncertain (48).

### 3.4 School and community food environments

Geospatial analyses indicate associations between built food environments and diet-related outcomes. Research in metropolitan Brazil indicates that over half of schools are situated within “food swamps” (defined as areas with a high density of UPF-selling convenience stores, fast-food outlets, and bars within a 250-meter radius) (49, 50). Furthermore, informal street vendors around schools serve as major conduits for cheap UPFs, reflecting deeper urban structural inequities (51). Cross-sectional evidence has associated combined residential and school food-swamp exposure with a higher prevalence of overweight and obesity (50).

Concerns about school food environments have been reported across diverse settings. In Soweto, South Africa, over half of the schools and their surrounding peripheries are saturated with SSB outdoor advertising and points of sale (52). Near elementary schools in Tehran, Iran, snacks utilizing child-targeted marketing (e.g., potato chips, extruded snacks) are strategically placed at store entrances and frequently flag high salt/sugar content via nutritional profiling (53). Moreover, socially deprived communities (“food deserts”) may have limited access to fresh produce retailers, constraining food choices for some low-income families (54–56). This high exposure to unhealthy built environments is recognized across Europe as a core upstream determinant stratifying childhood obesity by socioeconomic status (57).

## 4 Assessment tools for dietary inflammatory potential and the neurodevelopmental perspective

### 4.1 DII and C-DII: quantifying overall inflammatory burden

The DII estimates the pro-inflammatory potential of dietary intake from literature-derived weights for nutritional components associated with pro-inflammatory (e.g., CRP, IL-6, TNF-α) and anti-inflammatory cytokines (12). The C-DII adapts this dietary scoring approach for children; neither the DII nor C-DII directly measures systemic inflammation. Adolescent observational studies report associations between higher dietary inflammatory scores and biomarkers such as C-reactive protein (CRP), supporting evaluation of these biomarkers as external validation outcomes rather than as components of the dietary score (58). Studies report that dietary patterns high in UPFs or refined carbohydrates and low in dietary fiber are often associated with higher DII scores (i.e., highly pro-inflammatory diets) (59).

### 4.2 Impairment of neuropsychiatric and cognitive development

Maternal and childhood inflammatory exposures have been examined in relation to neurodevelopment, but evidence differs by developmental window and study design. Recent large-scale epidemiological investigations report an association between DII and pediatric neuropsychiatric disorders (NPDs) (14). Observational evidence associates higher UPF intake with metabolic outcomes, poorer psychological wellbeing, and mood symptoms in adolescents, including analyses adjusted for sedentary behavior (60). Furthermore, a higher dietary inflammatory score has been associated with lower academic performance in children; this finding does not establish that diet-induced inflammation caused the observed cognitive differences (61).

Interpretation of neurodevelopmental associations requires attention to reverse causality. Sensory sensitivities, restricted food repertoires, selective eating, and preferences for predictable or highly palatable foods may increase reliance on UPFs among some children with ADHD or autism spectrum disorder. Residual socioeconomic and behavioral confounding may also influence both diet and measured outcomes; therefore, causal direction remains unresolved.

#### 4.2.1 Transgenerational effects of maternal exposure

A meta-analysis comprising four European birth cohorts (the ALPHABET project) reported that higher maternal dietary inflammatory scores during pregnancy were associated with offspring internalizing symptoms (depression, anxiety), aggressive behaviors, and ADHD at ages 7–10 (62). Similarly, the INMA and RHEA cohorts in Spain reported associations between higher maternal DII and ADHD symptoms in preschool boys (63). Neuroimaging follow-ups further revealed that maternal dietary inflammatory scores during gestation were associated with offspring cortical gyrification patterns and later verbal IQ (64).

#### 4.2.2 Associations involving children's own diet

In children and adolescents, higher C-DII scores have been associated with ADHD (potentially in a non-linear U-shaped association) (65, 66), alongside more severe depressive and anxious symptoms (67). Some observational analyses have evaluated systemic inflammatory markers (e.g., CRP) and cardiometabolic factors (e.g., BMI z-score and waist circumference) as statistical mediators of associations between pro-inflammatory diets and mood disorders (67, 68).

#### 4.2.3 Association with autism spectrum disorder (ASD)

Recent studies reveal that children with ASD not only suffer from low dietary diversity and severe selective eating but also exhibit significantly higher C-DII scores, Systemic Immune-Inflammation Index (SII), and Systemic Inflammation Response Index (SIRI) compared to neurotypical peers (69). These cross-sectional differences may reflect dietary consequences of selective eating, correlated socioeconomic or behavioral factors, or bidirectional relationships; they do not show that diet causes ASD or ADHD. Accordingly, findings involving C-DII, SII, or SIRI should be interpreted as associations requiring longitudinal and mechanistic clarification.

## 5 Early-life UPF exposure and life-course metabolic trajectories

Longitudinal observational studies have examined whether pediatric UPF exposure is associated with subsequent metabolic and adiposity trajectories.

### 5.1 Accelerated adiposity rebound and altered life trajectories

The Avon Longitudinal Study of Parents and Children (ALSPAC) provides longitudinal observational evidence. Following over 9,000 children for more than a decade, the study indicated that children in the highest quintile of UPF consumption exhibited significantly steeper growth trajectories in Body Mass Index (BMI), fat mass index, body weight, and waist circumference from childhood into early adulthood compared to their low-consumption peers (70). These trajectories may be relevant to later cardiometabolic risk, although the observational design does not establish causality. A large Brazilian birth cohort also reported that higher UPF intake scores during the toddler years (2–4 years) were associated with changes in BMI z-scores and linear growth, supporting further evaluation of early-life dietary exposure and life-course metabolic trajectories (71). Longitudinal findings further indicate that anthropometric indicators in early life are predictive of low-grade inflammation and insulin resistance in adolescence, suggesting that pro-inflammatory patterns may emerge before adulthood (72).

### 5.2 Induction of “normal-weight obesity” (NWO)

Body-composition outcomes may differ among children with similar BMI values. Research involving Brazilian school-aged children revealed that high UPF intake and high C-DII scores were associated with higher odds of the “Normal-Weight Obesity (NWO)” phenotype—characterized by normal BMI but excessive body fat percentage (59). This implies that traditional BMI screenings may underestimate the potential metabolic risks associated with UPF intake, but longitudinal studies are needed to determine the temporal and biological interpretation of this association (73).

### 5.3 Infancy overweight and alterations in breast milk

Within extremely low-income households, associations with UPF exposure have been reported as early as infancy. Brazilian cohort studies highlight that frequent maternal UPF consumption, combined with short durations of exclusive breastfeeding (< 30 days), was associated with infant overweight in the first year of life (74). Furthermore, maternal obesity and dietary inflammatory potential have been associated with differences in breast-milk composition, increasing the pro-inflammatory fatty acid ratio (n-6/n-3) while depleting DHA and lutein, elements critical to neurodevelopment, thereby potentially disrupting early neural and visual maturation (75).

### 5.4 Exposomics and epigenetics: potential mechanisms of long-term dietary effects

Studies utilizing the “exposome” framework use metabolomic profiling to examine metabolic pathways associated with UPF exposure and obesity risk. ALSPAC metabolomic data show that high UPF intake in 7-year-olds correlates with 115 abnormal metabolic traits, including depleted levels of BCAAs and aromatic amino acids, alongside elevated citrate and glutamine; these metabolic differences are candidate intermediates rather than established mediators of fat accumulation (76).

Beyond transient metabolomic shifts, epigenetic regulation is a candidate mechanism for longer-term biological embedding, but direct pediatric and transgenerational evidence remains limited. Prospective cohorts and animal models have examined whether early dietary environments are associated with persistent DNA-methylation or histone-modification patterns. Possible cross-generational associations remain hypothesis-generating and require replication. For instance, the Lifeways cross-generation study showed that pre-pregnancy and prenatal maternal diet quality (e.g., HEI-2015 scores) was associated with differences in DNA methylation of genes linked to skeletal development and cell signaling (e.g., PLEKHM1, LUZP1) in 9-year-old offspring (77).

In pediatric cohorts, early maternal diet quality was associated with differences in offspring DNA methylation, particularly in core genes governing neurodevelopment (e.g., BDNF) and immune-inflammation (e.g., IL-6 related regions) (78). Additionally, recent meta-analyses of large EWAS involving multiple European child cohorts (e.g., HELIX, ALSPAC, Generation R) suggest that childhood UPF intake was associated with methylation differences at seven specific CpG sites (e.g., PHYHIP, NHEJ1, ATF7) largely involved in remodeling thyroid hormone and liver function pathways (7). These findings support further study of gene–environment relationships but do not establish a causal pathway to obesity (4).

Moreover, histone modifications may contribute to persistent inflammatory responses. A 2024 neurodevelopmental model suggested that maternal high-fat and pro-inflammatory dietary exposure may increase circulating cytokines (IL-6 and IL-1) and was associated with altered hippocampal H3K9me3 enrichment in offspring (79). The reported molecular changes were accompanied by altered receptor expression and behavioral outcomes in the model (79). A 2025 preclinical study of hepatic metabolic programming reported that H3K9me3 spatial conformational changes and abnormal gene expressions driven by early pro-inflammatory exposure (e.g., Western high-fat/high-sugar diets) remain incompletely reversible even after long-term transition to a healthy diet post-weaning (80). Together, these preclinical findings provide indirect biological plausibility; persistent neurodevelopmental, epigenetic, and transgenerational effects in children remain hypothesis-generating.

## 6 UPFs, the gut microbiome, and the systemic inflammatory network

Candidate pathways linking UPF-rich dietary patterns with inflammatory outcomes may involve gut microbial composition, intestinal barrier function, and systemic immune signaling (Figure 1). Specific inflammatory biomarkers may help characterize associations between dietary exposure and clinical outcomes by indicating chronic responses to dietary stressors (81). Figure 1 presents an evidence-informed conceptual model rather than an established pathophysiological cascade. It distinguishes dietary exposure, candidate microbial and barrier pathways, and observed or proposed outcomes. The links among these levels may be non-linear, and persistent epigenetic effects remain hypothesis-generating rather than established explanations of lifelong trajectories.

**Figure 1:**
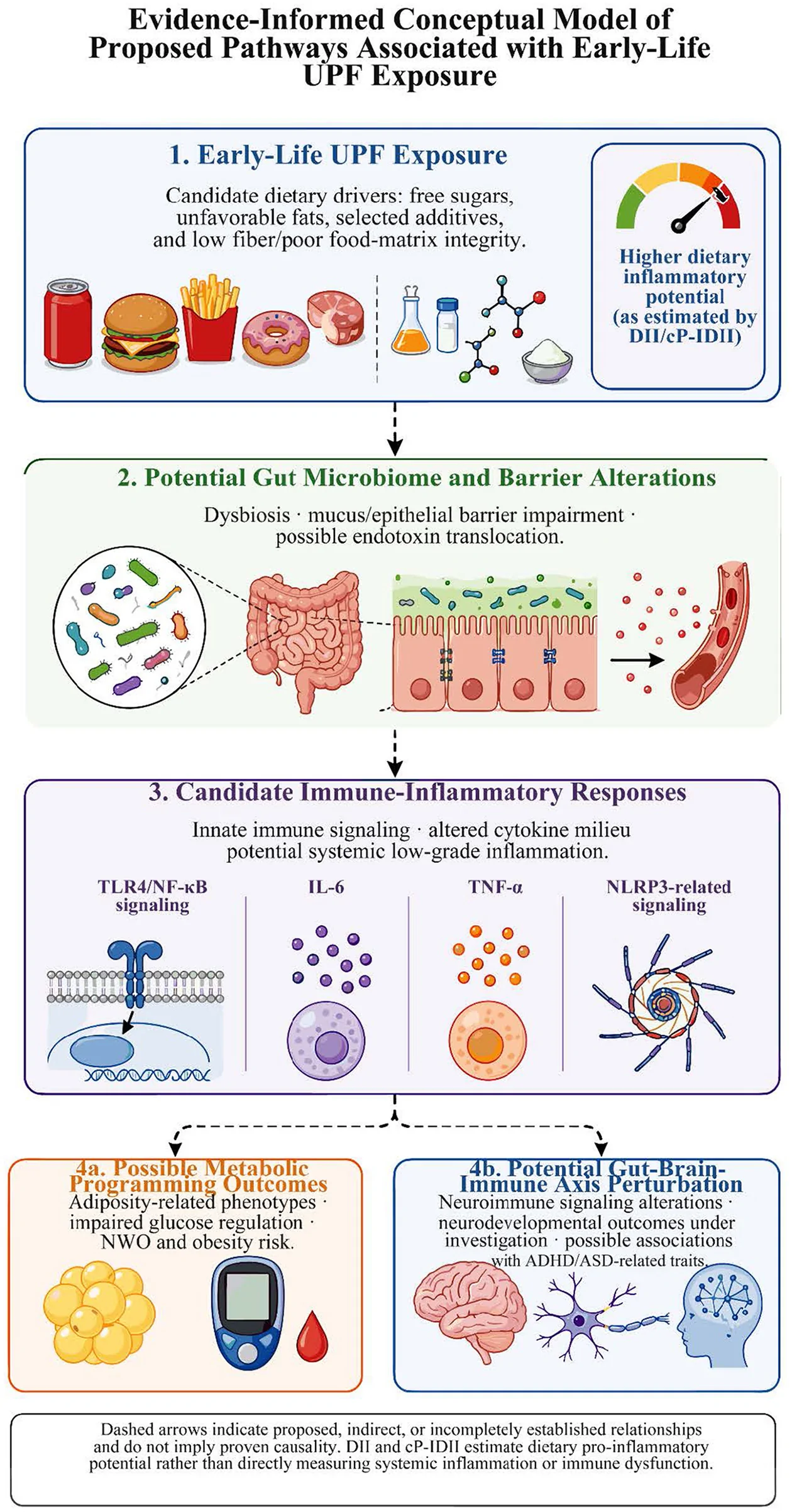
Evidence-informed conceptual model of the proposed pathways linking early-life ultra-processed food exposure to metabolic and neurodevelopmental outcomes. This figure is an evidence-informed conceptual model. Early-life exposure to ultra-processed foods may be characterized by high intakes of free sugars and unfavorable fats, selected food additives, low fiber content, and impaired food-matrix integrity. These dietary factors may contribute to alterations in gut microbial composition, intestinal mucus and epithelial barrier integrity, and possible endotoxin translocation. Subsequent changes in innate immune signaling, cytokine regulation, and inflammatory pathways, including TLR4/NF-κB and NLRP3-related signaling, may be associated with metabolic programming and perturbation of the gut–brain–immune axis. Dashed arrows indicate proposed, indirect, or incompletely established relationships and should not be interpreted as evidence of confirmed causality. The Dietary Inflammatory Index (DII) and the Pediatric-Specific Integrated Dietary Inflammatory Index (cP-IDII) estimate dietary pro-inflammatory potential rather than directly measuring systemic inflammation or immune dysfunction.

### 6.1 Compromised microbial diversity and the DI-GM index

Many UPF-rich dietary patterns are high in free sugars or fats and low in dietary fiber, features that may be associated with lower availability of substrates for butyrate-producing taxa. Pediatric studies report heterogeneous taxon-level differences rather than a consistent dysbiosis signature. Adult cross-sectional studies using the Dietary Index for Gut Microbiota (DI-GM) report associations between higher scores and selected inflammatory or clinical outcomes (82, 83). These adult findings provide indirect context and should not be generalized as causal pediatric effects.

### 6.2 Western and mediterranean dietary patterns

Ultra-processed food exposure generally occurs within broader dietary patterns. Western diets, characterized by high intakes of refined grains, added sugars, saturated fats, processed meats, and UPFs but low intakes of fiber and plant foods, may reduce microbiota-accessible substrates and favor less beneficial microbial profiles. In a cross-sectional study of 335 children and adolescents, a Western dietary pattern was associated with behavioral problems, lower levels of butyrate-producing species, and higher levels of selected potential pathogens; however, causality could not be established (84).

In contrast, the Mediterranean diet emphasizes plant foods, whole grains, fish, nuts, and olive oil while limiting refined and highly processed foods. Its higher fiber, unsaturated fat, and polyphenol content may support microbial diversity and short-chain fatty acid production. Greater adherence has been associated with higher microbial diversity and increased abundance of taxa such as Faecalibacterium and Prevotella, although the evidence is derived mainly from adult or mixed populations and remains heterogeneous (85). Replacing UPFs with minimally processed Mediterranean-style foods may therefore support microbial and metabolic health in children, but direct evidence that this pattern reverses UPF-related microbiome, immune, or neurodevelopmental alterations remains limited. It should be regarded as a health-promoting alternative rather than a proven antidote to UPF-related effects (84).

### 6.3 Food additives, artificial dyes, “leaky gut,” and endotoxemia

Beyond macronutrient imbalances, selected food additives have been investigated as potential contributors to intestinal-barrier changes. Emulsifiers and artificial sweeteners can degrade the intestinal mucus layer and downregulate tight junction protein expression, inducing “leaky gut”. Specifically, Allura Red (Red 40), a synthetic dye overwhelmingly prevalent in US pediatric foods, has been reported in animal models to alter microbial composition and markers of colonic inflammation and DNA damage (86, 87).

Previously, evidence of additive-induced mucosal damage was largely confined to animal or *in vitro* models (88), but recent adult randomized trials provide translational but indirect evidence for pediatric interpretation. A dietary intervention in healthy adults showed that just 11 days of consuming diets containing the emulsifier carboxymethylcellulose (CMC) led to diminished gut microbial diversity, significant depletion of short-chain fatty acids (SCFAs), and bacterial encroachment into the normally sterile inner mucus layer (89). Another human RCT involving five common emulsifiers reported changes in SCFAs after exposure to CMC or Polysorbate-80 (P80) (90), while carrageenan was associated with increased paracellular intestinal permeability (90), insulin resistance, and systemic low-grade inflammation (elevated CRP and IL-6) in overweight cohorts (91).

In modern pediatric diets, additives are present in many packaged staple foods. Screenings of commercial industrialized breads revealed that over 80% contain additives (like sodium stearoyl lactylate [SSL] and calcium propionate) that potentially mediate intestinal inflammation and dysbiosis (92–94). Preclinical studies have also examined possible intergenerational effects of additive exposure. Recent murine models revealed that maternal intake of emulsifiers (e.g., P80) was associated with offspring microbial and metabolic changes attributed to vertical microbial transmission (95). Barrier impairment, microbial-product translocation, and TLR4/NF-κB-related signaling constitute a candidate sequence; this continuous causal chain has not been demonstrated in children.

### 6.4 Pathological remodeling of the gut-brain axis

Alterations in gut microbial composition and intestinal permeability may influence neural outcomes through candidate gut–brain pathways, but the pediatric evidence is incomplete. Potential routes include circulating inflammatory signals, blood–brain-barrier interactions, enteroendocrine signaling, and vagal afferent pathways; their relative contribution to pediatric UPF associations is uncertain. Distributed along the intestinal epithelium, EECs directly “sense” luminal stimuli from high sugars, fats, and harmful additives. In a healthy gut-brain axis, EECs communicate with vagal afferents by releasing cholecystokinin (CCK) or glucagon-like peptide-1 (GLP-1) to maintain satiety and metabolic homeostasis. Preclinical work suggests that diet-related inflammation may alter enteroendocrine and vagal signaling (96). Candidate signals may reach brainstem, hypothalamic, limbic, or reward-related circuits (97, 98). Such mechanisms could contribute to eating behavior or neuroinflammatory signaling, but they do not establish that UPF exposure causes pediatric depression, anxiety, inattention, ADHD, or ASD. Accordingly, neurodevelopmental interpretations should distinguish observed associations from proposed mechanisms. Artificial sweeteners and emulsifiers have been investigated, primarily in adult or preclinical studies, as exposures that may affect microbial composition and barrier function. Peripheral inflammatory signaling and microglial activation are plausible intermediate pathways, but direct longitudinal pediatric evidence and causal direction remain limited (99, 100). Addressing these limitations, we propose the ‘Pediatric-Specific Integrated Dietary Inflammatory Index (cP-IDII),' an unvalidated three-dimensional research framework detailed in Figure 2.

**Figure 2:**
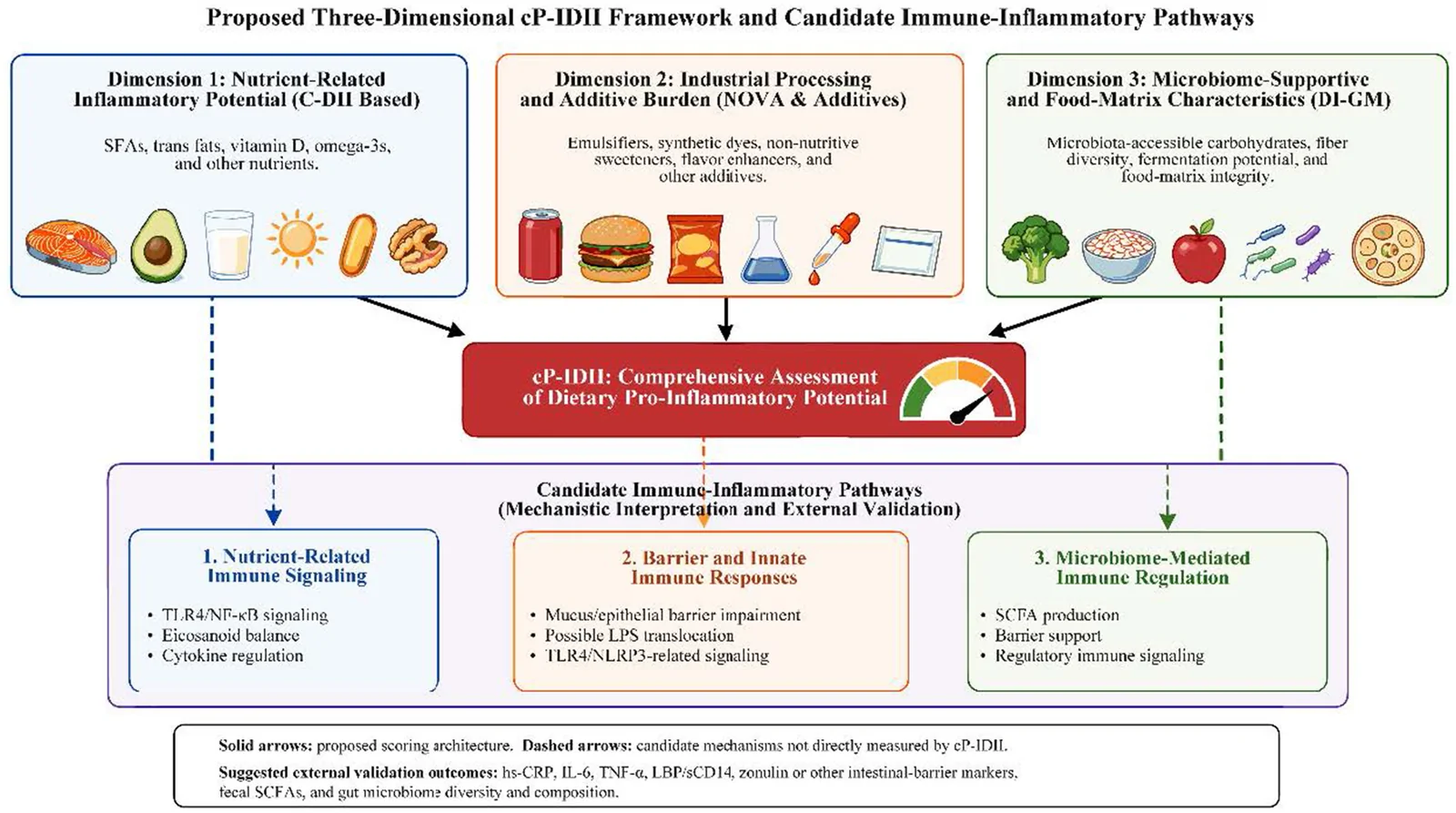
Proposed three-dimensional framework for the Pediatric-Specific Integrated Dietary Inflammatory Index (cP-IDII) and its candidate immune-inflammatory pathways. The cP-IDII integrates three complementary dimensions: nutrient-related inflammatory potential based on the conventional Dietary Inflammatory Index, industrial processing and additive burden based on NOVA classification and selected food additives, and microbiome-supportive and food-matrix characteristics. Solid arrows indicate the proposed scoring architecture through which the three dimensions contribute to the composite assessment of dietary pro-inflammatory potential. Dashed arrows indicate candidate mechanistic pathways that are not directly measured by the current index, including nutrient-related immune signaling, intestinal barrier impairment and innate immune activation, and microbiome-mediated immune regulation. Potential external validation outcomes include high-sensitivity C-reactive protein, interleukin-6, tumor necrosis factor-α, lipopolysaccharide-binding protein, soluble CD14, zonulin or other intestinal-barrier markers, fecal short-chain fatty acids, and measures of gut microbiome diversity and composition. These pathways remain conceptual and require validation in prospective human studies.

### 6.5 Potential immune-to-neuroinflammatory signaling pathways

An evidence-informed candidate sequence includes altered short-chain-fatty-acid signaling and barrier function, possible microbial-product translocation, TLR4/MyD88/NF-κB- and NLRP3-related signaling, cytokines such as IL-1β, IL-6, and TNF-α, and possible shifts in Th1, Th2, Th17, and Treg responses. Peripheral inflammatory, endocrine, vagal, and other neural routes may then transmit signals to the central nervous system. This sequence has not been demonstrated as a continuous causal pathway in children, and most detailed signaling evidence remains indirect.

A recent mechanistic review of microbiota-mediated immune modulation and gut–brain communication provides relevant indirect context (101), but it does not constitute evidence of pediatric UPF effects or causality.

## 7 Proposed cP-IDII framework and evidence-informed extensions

The DII and C-DII estimate nutrient-related dietary inflammatory potential, the EDIP uses empirical food-pattern weights, NOVA captures processing-related exposure, and the DI-GM summarizes dietary features associated with microbiome support. The proposed cP-IDII is intended to test whether reproducible nutrient and processing variables add information beyond these existing approaches. It is not a validated algorithm, and downstream biomarkers or microbiome measurements are not required score components.

Traditional DII models heavily weight the anti-/pro-inflammatory properties of natural macro- and micronutrients. However, in contemporary pediatric diets, food-matrix characteristics and selected industrial additives are candidate additional exposure domains whose independent effects remain uncertain. Recent cross-classification analyses indicate that the degree of processing and nutritional quality are distinct but frequently overlapping dimensions, supporting evaluation of both in assessment frameworks (102). Although recent simplified UPF scoring tools for toddlers have been developed, they do not jointly represent selected additive exposures and microbiome-supportive food-matrix features (103). The shift toward individualized dietary inflammatory assessment is gaining traction in adult populations, where novel indices like the Food inflammation scores of individuals (FISI) have been associated with sarcopenia in adult cross-sectional analyses (104). Addressing these limitations, we propose empirical development of an unvalidated, hypothesis-generating research framework: the “Pediatric-Specific Integrated Dietary Inflammatory Index (cP-IDII)”.

This integrated model encompasses three major dimensions (Table 1):

**Table 1:** Provisional scoring domains and evidence base for the unvalidated cP-IDII research framework.

Dimensions	Representative components	Scoring direction	Biological mechanism (condensed)	Evidence type	Evidence strength	Key limitations	Refs
Dim 1: nutrient burden (C-DII based)	SFAs, trans fats	Provisional positive (+)	Associated with TLR4-related and low-grade inflammatory pathways.	Pediatric observational and systematic-review evidence; adult evidence	Moderate for dietary-potential associations	Does not directly measure systemic inflammation; residual confounding	(8–11)
	Vit D, omega-3s	Provisional negative (–)	Associated with lower NF-κB signaling and cytokine expression.	Index-development and observational evidence	Moderate for score construction	Component effects are context-dependent	(11, 12)
Dim 2: industrial processing/additive	Emulsifiers (CMC, P80)	Provisional positive (+)	Candidate mucus-layer, permeability, and endotoxemia effects.	Adult RCT and mechanistic evidence	Indirect for children	Limited pediatric exposure and dose–response data	(89, 90)
	Synthetic dyes (red 40)	Provisional positive (+)	Preclinical changes in microbial and colonic markers.	Preclinical evidence	Low/indirect	No direct pediatric causal evidence	(86, 87)
	Non-nutritive sweeteners	Provisional positive (+)	Reported microbial and glucose-tolerance changes vary by compound.	Mainly preclinical and review evidence	Low/indirect	Dose, product, and formulation heterogeneity	(107, 109)
Dim 3: microbiome-supportive/food-matrix	MACs & fiber diversity	Provisional negative (–)	Supports SCFA production and barrier-related functions.	Adult observational and preclinical evidence	Indirect for children	Stool sequencing is not required for score calculation	(83, 115)
	Food matrix integrity	Provisional negative (–)	May slow nutrient absorption and influence oxidative stress.	Conceptual and cross-classification evidence	Hypothesis-generating	Operational measures and weights are not standardized	(16, 102)

This Table summarizes provisional scoring directions and the directness of supporting evidence. When additive concentrations are unavailable, fixed penalties should not be assigned; early models may compare presence/absence, consumption frequency, or product-category proxies and replace them with quantitative estimates when product-level data become available.

Traditional Macro-/Micronutrient Dimension (Based on traditional DII/C-DII): Evaluates the inflammatory burden of fatty acid profiles, vitamins, polyphenols, etc.

Industrial processing/additive dimension (incorporating NOVA classification): Evaluates candidate variables for industrial exposures, such as emulsifiers, artificial dyes (e.g., Red 40), non-nutritive sweeteners, and the content of dietary advanced glycation end products (AGEs) derived from thermal processing (105). Preclinical evidence suggests that some NNS (e.g., sucralose and aspartame) may have bacteriostatic effects (106) and have been linked to changes in gut microbial composition and SCFA production that may be accompanied by altered permeability and low-grade inflammation (107–109). Furthermore, studies have linked exogenous dietary AGEs with RAGE-related NF-κB and NLRP3 signaling, oxidative stress, and increased pro-inflammatory cytokine release (110–112). Recent pediatric research has associated high dietary AGE intake with impaired barrier function, Th1/Th2 imbalance, and food-allergy-related immune changes (113, 114). These findings support testing additive-related variables, but they do not justify fixed or severe penalty weights, particularly when concentrations and pediatric dose–response relationships are unavailable.

Microbiome-supportive/food-matrix dimension (Referencing DI-GM): Uses dietary fiber diversity, fermentation substrates, and food-matrix features that can be measured from dietary records or product databases; stool sequencing is not required to calculate the score (83). UPF-rich dietary patterns are often characterized by a low intake of microbiota-accessible carbohydrates (MACs) (115). Animal models suggest that microbiota-accessible carbohydrate supplementation may modify microbial composition, mucus-layer characteristics, and tight-junction expression (40). Preclinical studies also report associations with glial and neuroinflammatory outcomes (40). Whether these variables should receive protective weights, and at what magnitude, requires empirical estimation and sensitivity analysis.

This multidimensional framework is intended to generate testable measurement hypotheses. It does not directly quantify systemic inflammation, provide predictive biomarkers, or support clinical intervention decisions.

The following schematic equation illustrates candidate domains only; it is not a validated scoring algorithm. Additive, multiplicative, or threshold-based forms and all weights should be selected using empirical pediatric data rather than prespecified:


$$\begin{array}{l} cP-IDII=\overset{n}{\underset{i=1}{\sum }}\left(N_{i}\times W_{N_{i}}\right)+\overset{m}{\underset{j=1}{\sum }}\left(E_{j}\times P_{E_{j}}\right)-\overset{p}{\underset{k=1}{\sum }}\left(M_{k}\times B_{M_{k}}\right) \end{array}$$

Where *N*_*i*_ represents the intake level of traditional macro/micronutrients, and *W*_*N*_*i*__ is its established DII/C-DII weight; *E*_*j*_ represents industrial exposure factors (e.g., dyes, emulsifiers), and *P*_*E*_*j*__ is the newly introduced “Industrial Exposure Penalty Weight”; *M*_*k*_ represents dietary components beneficial to gut microecology (e.g., fiber diversity), and *B*_*M*_*k*__ represents the “Microbiome Protective Weight”.

Existing tools capture complementary nutrient, food-pattern, processing, or microbiome-supportive dimensions. The cP-IDII proposes testing whether a limited set of reproducible nutrient, processing/additive, and food-matrix variables can be combined without incorporating downstream microbiome or inflammatory outcomes into the exposure score.

This mathematical representation is an initial conceptual model. Candidate variables should be energy-adjusted and age-standardized. No fixed age-specific cutoffs are proposed at this conceptual stage; future development should compare continuous age-standardized scores with age-stratified reference distributions and empirically derived pediatric thresholds. Regional product-formulation databases may require recalibration. Empirical work should compare additive, multiplicative, and threshold-based forms, assess missing additive concentrations through sensitivity analyses, and estimate weights in longitudinal pediatric cohorts. External validation should precede any clinical, epidemiological, or public health use. The provisional scoring domains and supporting evidence are summarized in Table 1.

## 8 Public health policies and clinical interventions to address the pro-inflammatory environment

Within the exposure–mediator framework, front-of-pack labeling and reformulation primarily target product information and composition; school food standards reduce institutional UPF exposure; taxation and subsidies alter relative prices and affordability; marketing restrictions reduce child- and caregiver-directed promotional exposure; and additive-specific regulation may reduce exposure to compounds with plausible microbiome or barrier effects. These measures are complementary rather than interchangeable and should be evaluated for behavioral, biological, distributional, and equity outcomes.

Individual dietary education alone is unlikely to address the multilevel determinants of pediatric UPF exposure. A complementary policy portfolio can act on product information and composition, institutional exposure, relative affordability, marketing, and selected additive exposures (116). The complex interaction between commercial drivers and strategic policy solutions is summarized in Figure 3.

**Figure 3:**
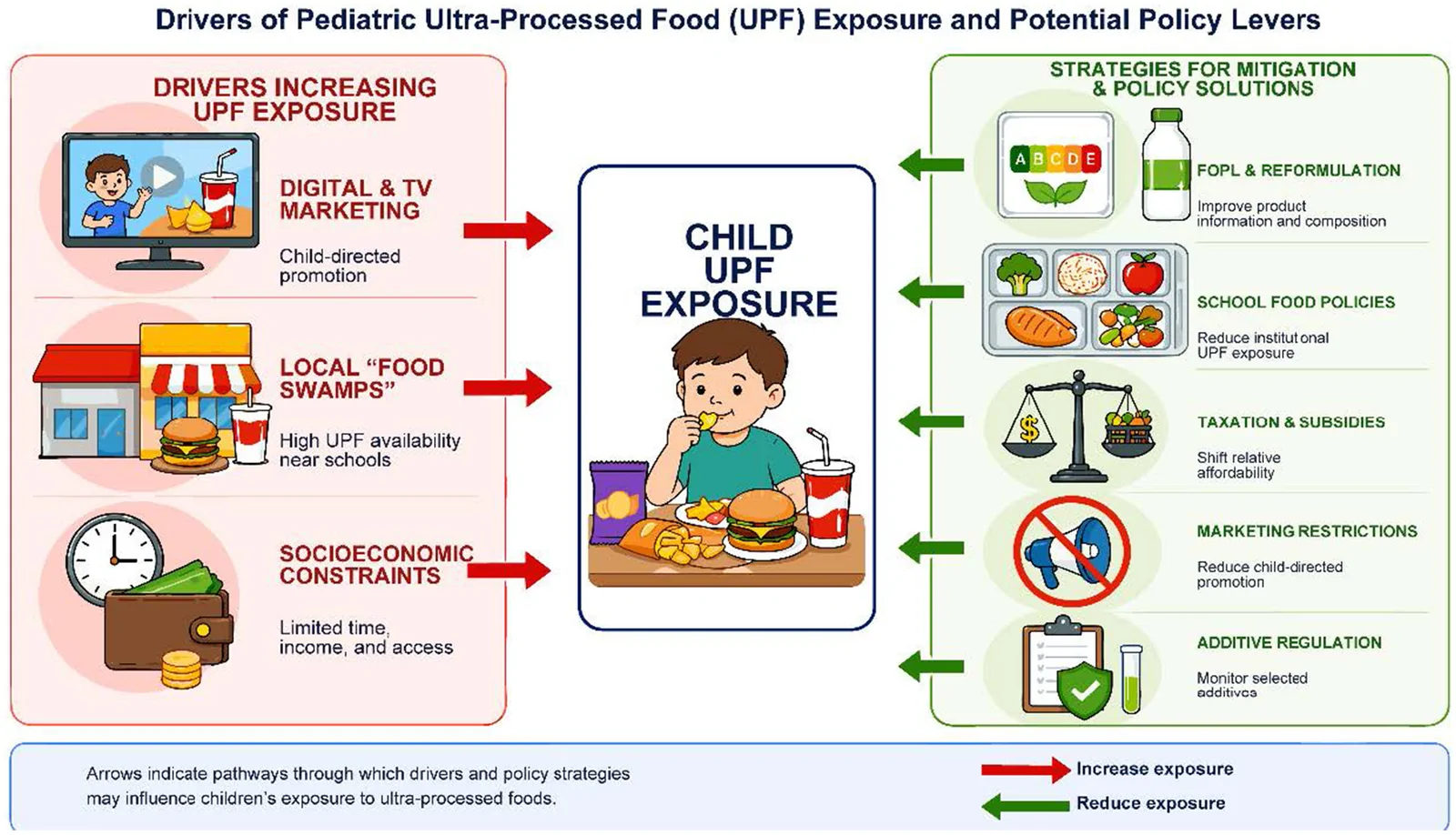
Socio-Ecological Determinants of Pediatric Ultra-Processed Food Exposure. The figure illustrates a socio-ecological framework in which pediatric UPF exposure is shaped by interacting determinants across multiple levels. Child-level factors include food preferences, developmental stage, and eating behavior. Caregiver and household influences include food purchasing practices, time constraints, nutrition knowledge, income, and home food availability. School and community factors encompass school meals, neighborhood food access, peer influences, and local food outlets. Commercial determinants include marketing, digital advertising, product formulation, packaging, pricing, and retail placement, whereas the policy environment includes food labeling, advertising regulation, school food standards, taxation, and subsidies. Bidirectional arrows indicate reciprocal interactions among these levels, emphasizing that pediatric UPF exposure is shaped by a dynamic system rather than by individual choice alone.

### 8.1 Advancing front-of-pack warning labels (FOPL) and product reformulation

Drawing on successes in Chile, Peru, and Mexico, mandatory “Front-of-pack warning labels” (FOPL) effectively modify consumer purchasing behaviors. The World Heart Federation (WHF) recommends that governments implement evidence-based FOPL (117). Qualitative research among Peruvian mothers reported strong recognition and comprehension of FOPL warnings and perceived effects on UPF purchasing frequency (118).

In real-world settings, FOPL policies may encourage product reformulation. Longitudinal data from Peru and Colombia report reductions in sugar or sodium content in top-selling products following warning label implementations (119, 120). Mexican regulations successfully prohibited misleading health claims on nearly 40% of junk food packaging (121). Compared to the Australian Health Star Rating (HSR) or the European Nutri-Score (which occasionally bestow “health halos” upon UPFs), the Chilean warning model may differ in sensitivity and effects on purchasing intentions (122–124). Furthermore, as NNS are increasingly used to evade sugar taxes, experts in Brazil and beyond are advocating for dedicated “NNS warning labels” to prevent secondary microecological and metabolic risks (125). Nonetheless, vigilance against policy dilution by food industry lobbying and public-private partnerships (PPPs) remains critical (126–128).

### 8.2 Reshaping the school food environment and universal free school meals

Schools are primary venues for pediatric caloric intake; therefore, the school food environment is an important and modifiable influence on dietary exposure. Natural experiments provide evidence relevant to universal free school meals (UFSM). Evaluations in the UK show that UFSM policies not only boost lunch participation but reduce in-school pediatric UPF intake by nearly 7%, with the most profound benefits observed in low-income demographics (129). Similarly, evaluations of the US Community Eligibility Provision (CEP) reported lower elementary-school obesity prevalence or BMI z-scores (130, 131), while simultaneously reducing household grocery expenditure on unhealthy foods by 5%−19% (132).

Longitudinal data indicate that food-swamp exposure and persistent availability of unhealthy competitive foods around schools are associated with increases in childhood obesity prevalence (133). Globally, a review of 193 nations found only 15% enforce national school meal nutritional restrictions (134). Detailed audits of school and university canteens reveal significant gaps between what is nutritionally intended and what is actually served and consumed, often characterized by high food waste and suboptimal nutritional profiles, indicating scope for structural improvements in institutional food environments (135, 136). Cities including New York and Copenhagen have extended school meal policies into planetary health strategies integrating sustainability and nutritional equity (137). Enacting strict regulations limiting UPF sales and advertising within and around schools may reduce institutional and marketing-related UPF exposure.

### 8.3 Strengthening marketing restrictions, taxation, and health equity

Confronting the escalating dual burden of malnutrition, taxation policies targeting SSBs and UPFs have entered core public health agendas globally (138). Simulation models in Chile indicate that taxes of 20%−30% on products exceeding nutrient thresholds could reduce purchasing and generate revenue to subsidize public health interventions (138, 139).

An intersectional perspective highlights that low-income and minority households may face time constraints and limited access to affordable fresh foods that shape UPF purchasing (140–142). Agent-based modeling suggests that policy effects may differ by social-transition stage; where UPF consumption is common among low-income populations, warning labels without concurrent price interventions could exacerbate health inequities (143). Consequently, concurrent with UPF taxation and pediatric marketing bans (34, 144), subsidy mechanisms may be paired with these policies to improve the accessibility and affordability of whole grains and fresh produce in marginalized communities (145, 146).

## 9 Current limitations, alternative explanations, and research priorities

### 9.1 Alternative explanations and measurement limitations

Associations between UPF intake and pediatric health may be partly attributable to higher energy density, faster eating rate, lower fiber intake, higher free-sugar or sodium content, socioeconomic disadvantage, and correlated health behaviors. Processing-related mechanisms may provide additional explanations, but their independent contribution remains incompletely established. NOVA categories contain nutritionally heterogeneous products, and exposure misclassification may occur when dietary records do not capture brand-specific formulation, portion size, or additive quantity. Evidence on individual additives often derives from experimental doses, adult participants, or preclinical models that may not represent real-world pediatric exposure.

Evidence linking UPF intake to ADHD, autism spectrum disorder, anxiety, depression, or cognitive outcomes is particularly vulnerable to reverse causality and residual confounding. Neurodevelopmental traits may influence food selectivity and reliance on convenient, predictable, or highly palatable products. These findings should therefore be interpreted as associations rather than evidence that UPFs cause neurodevelopmental disorders.

To reduce selective interpretation, claims were checked against the design and population of the cited studies, and adult or preclinical findings were not used as direct evidence of pediatric clinical effects.

### 9.2 Causal inference challenges and “reverse causality” risks

Current human evidence linking UPF exposure to pediatric neuropsychiatric disorders (e.g., ADHD, ASD) predominantly stems from cross-sectional or traditional longitudinal observational studies. Although animal gut–brain–inflammation models provide biological plausibility, reverse causality remains difficult to exclude in human populations. For instance, some children with ASD or ADHD exhibit sensory processing differences, restricted eating patterns, or differences in impulse control that may contribute to preferences for predictable or highly palatable foods. Distinguishing whether diet precedes neurodevelopmental outcomes, follows selective eating, or participates in a bidirectional relationship requires stronger longitudinal evidence. Future studies could combine genetically informed designs with long-term birth cohorts using repeated dietary and outcome measurements to clarify temporal sequence and causal direction. Comparative analyses across neurodevelopmental conditions should assess environmental, genetic, socioeconomic, and behavioral factors.

### 9.3 Policy intervention complexity and the “substitution effect” trap

At the policy level, isolated nutrient restrictions (e.g., free-sugar taxes or basic FOPL) may precipitate unintended “substitution effects”. In response to sugar-reduction policies, manufacturers may reformulate products with non-caloric sweeteners such as aspartame or with alternative additives to maintain sensory characteristics. However, as elucidated in our mechanistic sections, some substitutes have been associated with microbial or metabolic changes, although effects vary by compound, dose, and study design. Single-nutrient policies may therefore produce substitution effects and shift rather than eliminate relevant exposures. Consequently, future policy formulation and empirical development of frameworks such as the cP-IDII should examine a transition from “single-nutrient reductionism” to holistic evaluations of the “food matrix” and degree of processing. Policy evaluations could consider processing, additives, and potential microbial effects while monitoring reformulation and substitution. Future research and policy should also consider exposures among low-income families and evaluate interventions for affordability and equity.

## 10 Conclusion

UPFs contribute substantially to many pediatric and adolescent diets and warrant evaluation within the broader determinants of diet quality and energy intake. Pediatric observational studies most consistently report associations with poorer diet quality and less favorable adiposity or cardiometabolic trajectories. Processing-related characteristics, food-matrix disruption, and selected additives provide candidate additional pathways, but their effects independent of total energy intake and nutrient composition remain uncertain, particularly in children.

The proposed “pro-inflammatory imprint” is an evidence-informed, hypothesis-generating framework. Microbial composition, intestinal-barrier function, inflammatory signaling, and immune-to-neural communication are biologically plausible candidate pathways supported by varying combinations of pediatric, adult, and preclinical evidence. Persistent neurodevelopmental, epigenetic, or transgenerational effects remain particularly uncertain, and associations involving ADHD or ASD may also reflect reverse causality and residual confounding.

Caregiver decisions occur within household, commercial, institutional, cultural, and policy contexts. Front-of-pack labeling and reformulation, school food standards, taxation and subsidies, marketing restrictions, and additive-specific regulation act at different and complementary points in this system and should be assessed for substitution and equity effects. The cP-IDII remains an unvalidated research framework; empirical score development, regional calibration, and external validation are required before it can inform clinical, epidemiological, or public health decisions.
